# Influence of Oxygen Concentration on the Performance of Ultra-Thin RF Magnetron Sputter Deposited Indium Tin Oxide Films as a Top Electrode for Photovoltaic Devices

**DOI:** 10.3390/ma9010063

**Published:** 2016-01-20

**Authors:** Jephias Gwamuri, Murugesan Marikkannan, Jeyanthinath Mayandi, Patrick K. Bowen, Joshua M. Pearce

**Affiliations:** 1 Department of Materials Science & Engineering, Michigan Technological University, 1400 Townsend, Houghton, MI 49931, USA; jgwamuri@mtu.edu (J.G.); pkbowen@mtu.edu (P.K.B.); 2 Department of Materials Science, School of Chemistry, Madurai Kamaraj University, Ta mil Nadu, Madurai 625 019, India; marikannan.mku@gmail.com (M.M.); jeyanthinath.mayandi@gmail.com (J.M.); 3 Department of Electrical & Computer Engineering, Michigan Technological University, 1400 Townsend, Houghton, MI 49931, USA

**Keywords:** transparent conducting oxide, indium tin oxide, plasmonics, wet etching, photovoltaics, optics

## Abstract

The opportunity for substantial efficiency enhancements of thin film hydrogenated amorphous silicon (a-Si:H) solar photovoltaic (PV) cells using plasmonic absorbers requires ultra-thin transparent conducting oxide top electrodes with low resistivity and high transmittances in the visible range of the electromagnetic spectrum. Fabricating ultra-thin indium tin oxide (ITO) films (sub-50 nm) using conventional methods has presented a number of challenges; however, a novel method involving chemical shaving of thicker (greater than 80 nm) RF sputter deposited high-quality ITO films has been demonstrated. This study investigates the effect of oxygen concentration on the etch rates of RF sputter deposited ITO films to provide a detailed understanding of the interaction of all critical experimental parameters to help create even thinner layers to allow for more finely tune plasmonic resonances. ITO films were deposited on silicon substrates with a 98-nm, thermally grown oxide using RF magnetron sputtering with oxygen concentrations of 0, 0.4 and 1.0 sccm and annealed at 300 °C air ambient. Then the films were etched using a combination of water and hydrochloric and nitric acids for 1, 3, 5 and 8 min at room temperature. In-between each etching process cycle, the films were characterized by X-ray diffraction, atomic force microscopy, Raman Spectroscopy, 4-point probe (electrical conductivity), and variable angle spectroscopic ellipsometry. All the films were polycrystalline in nature and highly oriented along the (222) reflection. Ultra-thin ITO films with record low resistivity values (as low as 5.83 × 10^−4^ Ω·cm) were obtained and high optical transparency is exhibited in the 300–1000 nm wavelength region for all the ITO films. The etch rate, preferred crystal lattice growth plane, d-spacing and lattice distortion were also observed to be highly dependent on the nature of growth environment for RF sputter deposited ITO films. The structural, electrical, and optical properties of the ITO films are discussed with respect to the oxygen ambient nature and etching time in detail to provide guidance for plasmonic enhanced a-Si:H solar PV cell fabrication.

## 1. Introduction

Solar photovoltaic (PV) based electricity production is one of the significant ecofriendly methods to generate sustainable energy needed to mitigate the looming global energy crisis [1]. Despite technical improvements [2] and scaling [3], which have resulted in a significant reduction in crystalline silicon (c-Si) PV module costs, for continued PV industry growth [4,5], PV costs must continue to decline to reach a levelized cost of electricity [6] low enough to dominate the electricity market. One approach to reduced PV costs further is to transition to thin film PV technology [7]. Hydrogenated amorphous silicon (a-Si:H) based PV [8] have shown great potential for large scale [9] sustainable commercial production due to lower material costs and use of well-established fabrication techniques [10,11]. However, there is need to improve the efficiency of a-Si:H PV devices if they are to become the next dominant technology for solar cells commercialization. One method to improve a-Si:H PV performance is with optical enhancement [12]. Recent developments in plasmonic theory promise new light management methods for thin-film a-Si:H based solar cells [13,14,15,16,17,18,19,20,21,22,23]. However, previous work has shown these plasmonic approaches require the development of ultra-thin, low-loss and low-resistivity transparent conducting oxides (TCOs) [24]. Tin doped indium oxide (ITO), zinc oxide (ZnO) and tin oxide (SnO_2_) are the three most important TCOs and are already widely used in the commercial thin film solar cells [25]. In addition, aluminum-dope zinc oxide (AZO) and fluorine-doped tin oxide (FTO) are among the other most dominant TCOs in various technological fields particularly the optoelectronic devices industry where TCOs have proved indispensable for applications such as photo electrochemical devices, light emitting diodes, liquid crystal displays and gas sensors [26,27]. ITOs can be prepared by direct current (DC) and radio frequency (RF) magnetron sputtering, electron beam evaporation, thermal vapor evaporation, spray pyrolysis, chemical solution deposition, and sol gel methods [28,29,30,31,32,33,34]. RF magnetron sputtering can be used to control the electrical and optical properties of the ITO thin films and is heavily used in industry [35]. 

Recent work by Vora *et al.* has emphasized the need for ultra-thin ITO top electrodes with low resistivity and high transmittances in the visible range of the electromagnetic spectrum as a prerequisite for the commercial realization of plasmonic-enhanced a-Si:H solar cells [36]. However, research by Gwamuri *et al.* has demonstrated that fabricating ultra-thin ITO films (sub-50 nm) using conversional methods presented a number of challenges since there is a trade-off between electrical and optical properties of the films [37]. It was evidenced from their results that electrical properties of RF sputter deposited sub-50 nm ITO films degraded drastically as their thickness is reduced, while the optical properties of the same films were seen to improve greatly [37]. To solve this problem, a novel method involving chemical shaving of thicker (greater than 80 nm) RF sputter deposited films was proposed and demonstrated [38]. Building on the promise of that technique, this study seeks to further understand the effect of oxygen concentration on the etch rates of RF sputter deposited ITO films and the impact on the TCO quality as a top electrode for PV devices. A detailed understanding of the interaction of all critical parameters, which determines the quality of ultra-thin ITO will help create even thinner layers with good quality to allow more finely tuned plasmonics resonances. ITO films were deposited using four different oxygen concentrations (0 sccm, 0.4 sccm, 1.0 sccm), annealed in air at 300 °C for 30 min and then etched for four different times (1, 3, 5 and 8 min) to establish the effect of oxygen on etch rates. These materials were characterized by X-ray diffraction (XRD), atomic force microscopy (AFM), Raman Spectroscopy, 4-point probe (4PP), and variable angle spectroscopic Ellipsometry (VASE). In addition, the thin films were investigated for candidates as acid-resistant TCOs for encapsulation of PV devices, which may reduce device processing steps and fabrication costs of completed modules in the future. The results are presented and discussed.

## 2. Materials and Methods

### 2.1. ITO Fabrication Process

ITO films were grown on (100) prime silicon substrates with a 98 nm thermally grown oxide, and on glass substrates using a 99.99% 100 mm diameter pressed ITO (SnO_2_:In_2_O_3_ 10:90 wt%) target. Before the deposition the substrates were ultrasonically cleaned in isopropanol and in DI water for 15 min and dried using N_2_ atmosphere. The sputtering chamber was initiated to a low 10^−7^ Torr base pressure and the pressure was maintained at 7.5 × 10^−3^ Torr. The distance between the target and substrates was kept constant at 75 mm. As a standard procedure, the target was pre-sputter cleaned at a power of 150 W, whereas the sputter deposition of the films was performed at 100 W. The argon gas flow rate was fixed at 10 sccm and the oxygen gas flow was varied such as 0, 0.4 and 1.0 sccm with sputter rate of 8–12 nm per minute. The sputter rate was seen to decrease with increase in oxygen flow rate. After deposition, ITO films were annealed at 300 °C for 30 min in air. ITO/Si films were subjected to the etching process using a standard chemical etchant mixture of HCl: HNO_3_:H_2_O (1:1:5) volume ratio. All the etching was performed at room temperature, resulting in a slow and controlled etch rate for the Si/SiO_2_ films. Finally, the etched samples were thoroughly rinsed in DI water and dried under the nitrogen environment. This methodology was adapted from the previous study by Gwamuri *et al.*, 2015 [37].

The ITO films processed under different argon-oxygen ambient were chemically etched and characterized using various tools. The structural analyses of the ITO films were carried out using X-ray diffraction (XRD-Scintag-2000 PTS, Scintag Inc., Cupertino, CA, USA). Raman spectra for the ultra-thin film samples were measured at room temperature using Jobin-Yvon LabRAM HR800 Raman Spectrometer (Horiba Scientific, Edison, NJ, USA) with the excitation wavelength of 633 nm and the resolution is about ~0.1 cm^−1^. Sheet resistance of the samples was characterized using four point probe station consisting of ITO optimized tips with 500 micron tip radii set to 60 grams pressure and an RM3000 test unit from Jandel Engineering Limited, Kings Langley, UK. The optical transmission and thickness of the films was determined using variable angle spectroscopic ellipsometry (UV-VIS V-VASE with control module VB-400, J.A. Woollam Co., Lincoln, NE, USA). Surface roughness was evaluated using a Veeco Dimension 3000 atomic force microscope (Veeco, Oyster City, NY, USA) operated in tapping mode with Budget Sensors Tap300Al-G cantilevers (Innovative Solutions Bulgaria Ltd., Sofia, Bulgaria). It should be noted that transmittance data was measured for ITO on sodalime glass (SLG) substrate and all the rest of the data was on ITO on Si/SiO_2_ substrate.

### 2.2. Chemical Shaving: Wet Etching

In this present work, the oxygen 0, 0.4 and 1.0 sccm deposited ITO films were used for the etching process for 1, 3, 5 and 8 min, respectively. The annealed ITO/Si samples are etched at room temperature using HCl:HNO_3_:H_2_O (1:1:5) combination and the resistivity and thickness of the films were checked for 1, 3, 5 and 8 min etched films. For the 0 sccm ITO films, the thickness of the film was changed from 70 to 44 nm for 1 to 5 min etching time. Similarly the 0.4 sccm films thickness changed from 89 to 47 nm and 84 to 22 nm for 1.0 sccm films. The decrement of thickness was reflected in the resistivity values. The chemical reaction of the HCl and HNO_3_ etching reactions are as follows [39]:

(1) In_2_O_3_ + 2HCl → 2InCl + H_2_O + O_2_ (∆H)

(2) In_2_O_3_ + 12HNO_3_ → 2In (NO_3_)_3_ + 6NO_2_ + 6H_2_O

## 3. Results

### 3.1. Structural Analysis

#### 3.1.1. XRD Analysis

**Figure 1:**
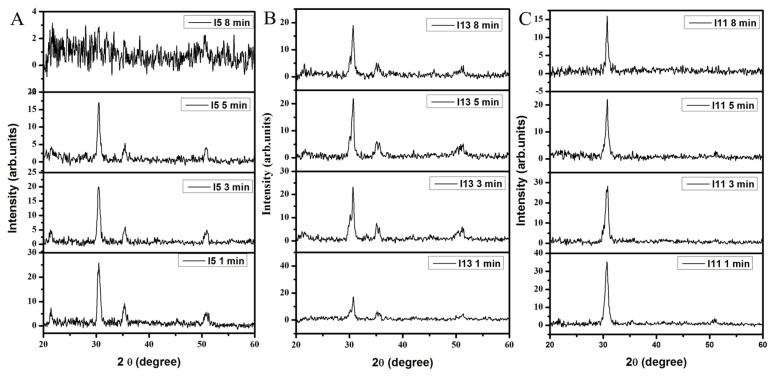
XRD pattern for ITO films deposited under different oxygen ambient conditions and etched for 1, 3, 5 and 8 min: (**A**) 0 sccm oxygen; (**B**) 0.4 sccm oxygen; (**C**) 1.0 sccm oxygen. Argon flow rate was maintained at 10 sccm for all materials.

XRD results for the ITO films deposited using different oxygen concentrations (0 sccm, 0.4 sccm, 1.0 sccm), annealed in air at 300 °C for 30 min and then etched for different times (1, 3, 5 and 8 min) are shown in Figure 1.

In addition to that the peak shown at (222), (400) and (440) reflections are indexed to be cubic indium oxide (JCPDS No: 06-0416) [40]. All the films have a polycrystalline nature with stronger (222) reflection. No other tin phases could be identified from the cubic indium tin oxide. Normally the 30% of Sn is needed to exhibiting the SnO_2_ diffraction lines in ITO. The (222) and (400) plane is ascribed for oxygen efficient and deficient nature of ITO films [41]. The effect of the oxygen flow rate on the peak intensity of the ITO films is clearly shown in the XRD spectrum. There is a general increase in the peak intensities with increased oxygen flow rate. Similarly the reflections such as (211), (400) and (440) are due to the minimum oxygen concentration in the sputter chamber. These planes are absent in the XRD pattern of ITO film processed in an oxygen-rich (1.0 sccm) environment. There is a strong evidence that for the highest oxygen ambient (1.0 sccm), (222) is the preferred growth orientation for RF sputter deposited ITO films. Varying the oxygen concentration will result in changing the preferred growth orientation of the films to other crystal lattice planes such as the (211), (400) or (440). The intensity ratios are strongly dependent on the critical level of In^3+^ and O^2−^ pairs and the pairs’ density is different for different etching periods of time [42]. The presence of high oxygen concentration induce the In-O bonding networks formation and promote growth of the (222) crystal lattice planes.

During the etching, ITO films are reduced to In–Cl and In-(NO_3_)_3_ resulting in the change in crystallinity of films etched for different periods of time. The structural parameters such as d spacing, lattice constants, net lattice distortion and grain sizes are estimated and listed in Table 1 in comparison to data from the Joint Committee on Powder Diffraction Standards (JCPDS)/International Centre for Diffraction Data (ICDD) database.

The etching process also distorts the ITO structural long-range order, which has an impact on the opto-electronic properties of the films. The grain size of films did not change even after etching for 8 min., particularly for ITO films processed in an oxygen deficient environment. During the etching process the excess weakly bound oxygen atoms are removed from the ITO surfaces exposing layers with different grain sizes. The ITO structure distortion due to etching for longer periods of time (8 min) can be seen from the XRD spectra shown in Figure 1. There was however no evidence of ITO film for the results shown in Figure 1A after they were etched for 8 min. There is evidence of decreased crystallinity for the rest of the ITO films (Figure 1B,C) as the oxygen atoms are stripped from the In–O network by the HCl and HNO_3_. 

**Table 1:** Structural parameters of ITO sputtered films with 0, 0.4 and 1.0 sccm oxygen and etched at 1, 3, 5 and 8 min.

Oxygen Flow Rate (sccm)	Etching Time (min)	D Spacing (222) (Å)	Lattice Constant (222) (Å)	Net-Lattice Distortion	Grain Size (222) (nm)
Standard JCPDS for ITO 06-0416	–	2.921	10.1180	–	–
0	1	2.932	10.1552	–0.0036	16
	3	2.934	10.1629	–0.2970	16
	5	2.934	10.1629	–0.2885	17
	8	–	–	–	–
0.4	1	2.908	10.0731	–0.0075	31
	3	2.912	10.0869	–0.0157	25
	5	2.914	10.0954	–0.0153	23
	8	2.914	10.0954	–0.0169	20
1.0	1	2.917	10.1059	–	13
	3	2.913	10.0915	–0.2828	13
	5	2.911	10.0845	–	14
	8	2.908	10.0764	–	19

#### 3.1.2. AFM Analysis

**Figure 2:**
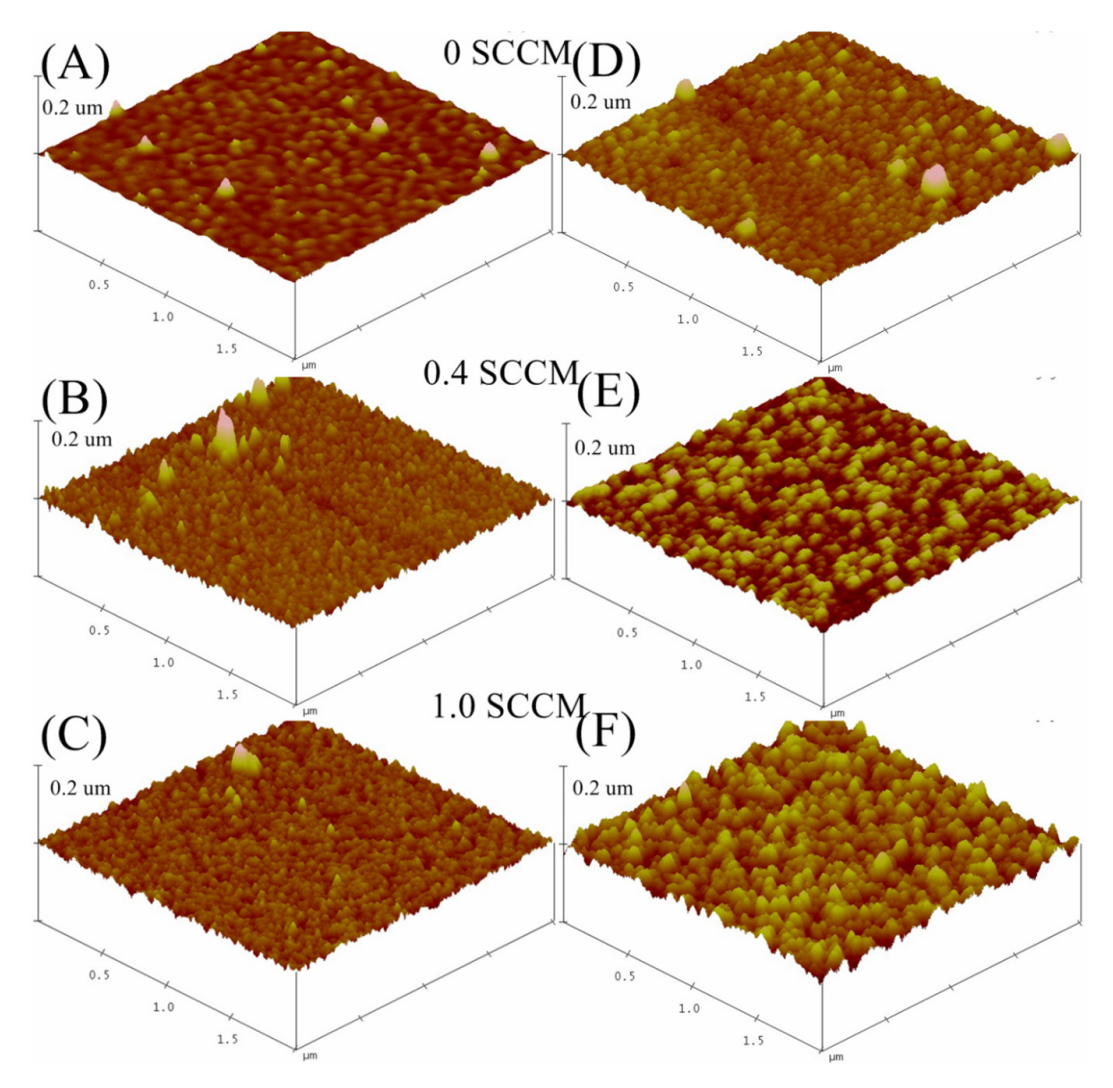
Surface topology image for 2 µm × 2 µm × 0.2 µm of the ITO film deposited under various oxygen environments: (**A**) 0 sccm oxygen; (**B**) 0.4 sccm oxygen; (**C**) 1.0 sccm oxygen, and etched for 8 min; (**D**) 0 sccm oxygen; (**E**) 0.4 sccm oxygen and (**F**) 1 sccm oxygen. (**A**–**C**) etched for 1 min and (**D**–**F**) films etched for 8 min. The etching was performed at room temperature.

Figure 2 shows the AFM surface topology of the ITO films deposited under three different oxygen environments and etched for 1 min and 8 min. 

Figure 2A,B shows the ITO films deposited in an oxygen deficient ambient and etched for 1 and 8 min, respectively. Spherical sized grains are clearly visible in all AFM images presented in Figure 2. There is variation of surface roughness of the films with both oxygen flow rate and etching time of the ITO films. The minimum value of surface roughness of 0.65 nm was measured for ITO films sputtered using 0.4 sccm oxygen flow rate and etched for 1 min, while a maximum surface roughness value of 8.9 nm was observed for films processed at 1.0 sccm oxygen flow rate and etched for 1 min. There was a slight increase in roughness with etching time observed for 0 sccm and 0.4 sccm ITO film, for etching times 1 min to 8 min. However, the 1.0 sccm films, showed the greatest variation in surface roughness even after 1 min etching process. Generally, the surface roughness of the films are observed to increase when the oxygen gas concentration is increased during processing. 

#### 3.1.3. Raman Spectroscopy

**Figure 3:**
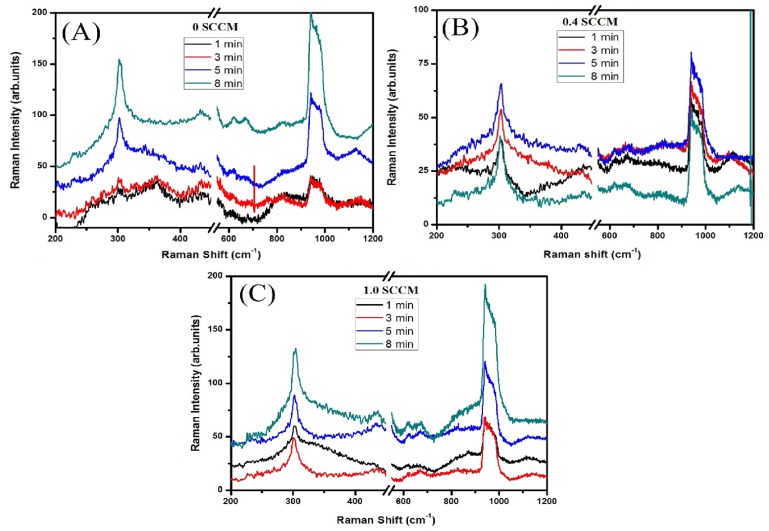
Raman spectra for the ITO films deposited under various oxygen concentrations and etched for 1, 3, 5 and 8 min., respectively. (**A**) 0 sccm; (**B**) 0.4 sccm; (**C**) 1.0 sccm.

Figure 3 shows the Raman spectrum for ITO deposited at various oxygen compositions and etched at 1, 3, 5 and 8 min respectively. Raman spectroscopy is used to determine the structural conformations of the materials. Group theory predicts the Raman modes for cubic indium oxide, such as 4Ag (Raman), 4Eg (Raman), 14Tg (Raman), 5Au (inactive), and 16Tu (infrared) modes [43]. The modes observed are at 303, 621 and 675 cm^−1^ for all the films. Noticeable modes are exhibited at 302 and 621 for Eg and In–O vibrational mode [44]. The observed Raman modes in Figure 3 are in good agreement with previous reported results [40]. There are no other additional modes observable for the SnO and SnO_2_ structures. In addition to that the broad band shown at 976 to 1013 cm^−1^ for all the etched films and it was not unassignable. The peak appeared at 1132, 1112, 1097 and 1120 cm^−1^ for 0, 0.4 and 1.0 sccm ITO etched films. These peaks are reported in the commercially ITO films [45]. The Raman results are correlated with XRD results. No other mixed phases were observed in the Raman spectrum indicating that etching process had no or little effect on the ITO structure.

### 3.2. Resistivity

The electrical properties of the different oxygen ambient deposited and etched ITO films were measured using a four point probe. The sheet resistance values of the ITO films are changed with respect to the oxygen ambient nature and etching time and are summarized in Table 2. 

**Table 2:** Electrical and optical parameters of ITO films deposited under various oxygen compositions and etched for 1, 3, 5 and 8 min.

Oxygen Flow Rate (sccm)	Etching Time (min)	Sheet Resistance (Ω/square)	Thickness (nm)	Resistivity (Ω·cm)	Transmission (%)
0	1	83.28	70	5.83 × 10^−4^	76.29
	3	103.47	59	6.11 × 10^−4^	93.98
	5	209.49	44	9.22 × 10^−4^	90.27
	8	–	–	–	100
0.4	1	209.02	89	1.86 × 10^−3^	91.45
	3	194.23	88	1.71 × 10^−3^	85.26
	5	240.08	85	2.04 × 10^−3^	84.85
	8	326.9	47	1.54 × 10^−3^	83.71
1.0	1	1000	84	8.4 × 10^−3^	90.96
	3	2000	62	1.24 × 10^−2^	89.25
	5	2400	50	1.20 × 10^−2^	100
	8	7350	22	1.62 × 10^−2^	100

From the obtained results, the minimum sheet resistance was observed for ITO deposited using argon ambient (0 sccm oxygen) and etched for 1 min. However, the same films exhibited the worst transmittance of about 76%. During processing in an argon rich environment, the bombardment by argon neutrals creates dangling bonds in the substrates and created the oxygen vacancies in the ITO films [46]. The argon environment (10 sccm) (*i.e.*, the oxygen deficient environment) promotes oxygen vacancies that enhance electrical resistivity while degrading the optical properties of the films. This is reflected in the XRD spectra, where the (400) and (440) lattice planes are enhanced for ITO films processed in low oxygen (0 sccm and 0.4 sccm) environments. Hence, the 1.0 sccm deposited films in which the (222) lattice plane is dominant, showed a higher electrical resistivity compared to the other films. The resistivity of the films are highly dependent on the film thickness, which is a function of the etching time. Increasing the etching time decreases the thickness of the films, and, hence, the electrical properties while improving optical properties. 

### 3.3. Transmittance

**Figure 4:**
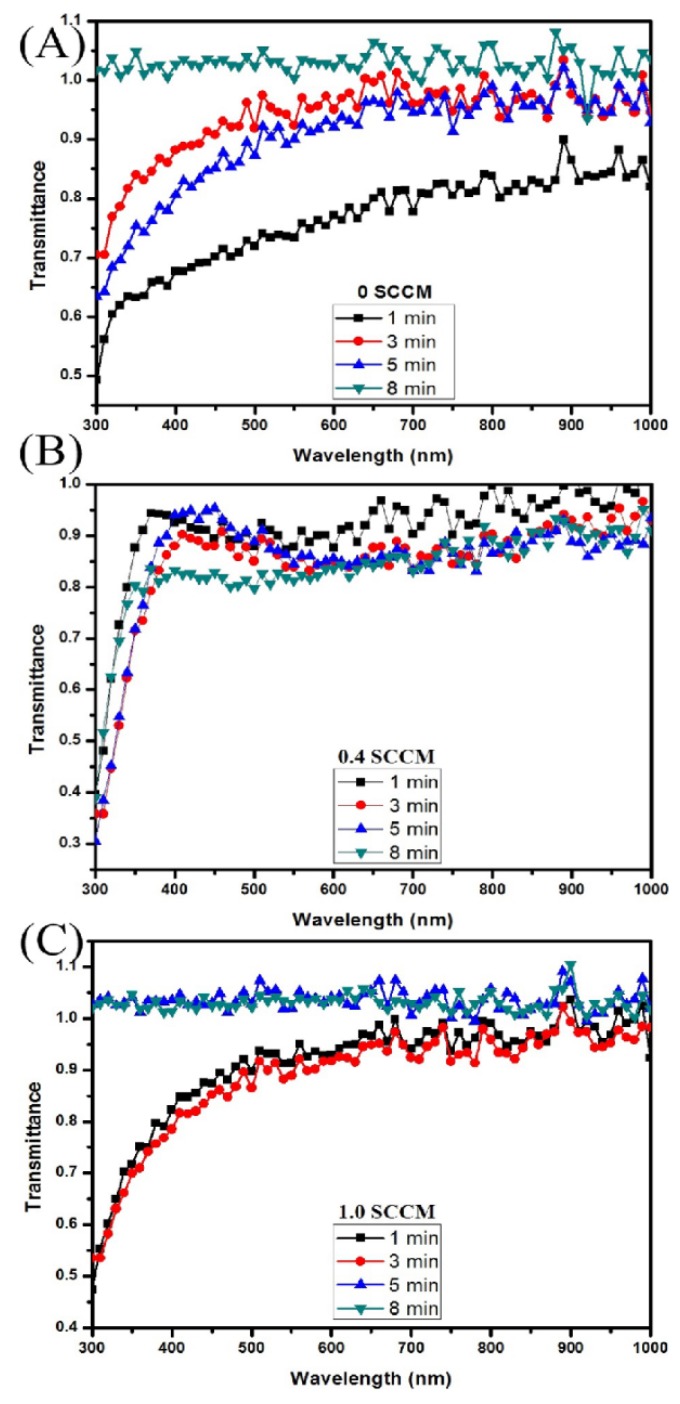
Optical transmission spectrum for the RF sputter deposited ITO films at different oxygen compositions and etched for min (**A**) 0 sccm; (**B**) 0.4 sccm; and (**C**) 1.0 sccm.

Optical transmittances of the ITO films on glass substrates are recorded from 300 to 1000 nm at room temperature and shown in Figure 2. All the films exhibited the highest average optical transmittance in the higher wavelength range. The highest optical transmittance is attained for 0sccm oxygen ITO film etched for 8 min with and average etch rate of 5.2 nm/min (for 5 min etch) and the 1.0 sccm films etched for 5 and 8 min with average etch rates of 5.25 and 7.75 nm/min, respectively. The results are summarized in Table 2. The thickness of the film is an important parameter for determining both electrical and optical properties of the ITO films. In this work, the thickness was quantified using spectroscopic ellipsometry measurements and is shown in the Table 2. For transmittance measurements, the ITO films were deposited on SLG substrates. The SLG transmittance is measured and used as baseline data. All ITO films transmittance data involve baseline subtraction, hence 100% transmittance means that all of the ITO film has been etched off. The etch rates were much faster for the ITO on glass such that all the film was etched-off after an 8-min etch (Figure 4A), and 5 and 8 min (Figure 4C).

The results show a direct correlation between the oxygen concentration and the optical transmittances of the films and an inverse relationship with the electrical conductivity of the ITO films. These results are in agreement with observation from previous studies [47,48]. Figure 4A for 8 min etch, and Figure 4C for 5 and 8 min etch showed a transmittance cut-off wavelength around 350 nm indicating the absorption edge. 

## 4. Discussion

The results presented provide further insight on the interaction of the most common fabrication variables that influence the electrical and optical properties of ITO films for PV and other opto-electronic applications. There is evidence of a strong correlation between oxygen concentration and both the resistivity and transmittances of RF sputter deposited ITO films. The processing conditions have a strong bearing on the structure of the ITO films. The (222) lattice planes are preferred in films grown in oxygen rich ambient whilst more lattice planes; (211), (222), (400), and (440) are observed in films grown under less oxygen or oxygen deficient conditions. Wan *et al.* have reported the (211), (400) and (440) planes reflections as being associated with ITO films processed under high RF power in an oxygen deficient atmosphere [35]. From the structural analysis, the exhibited (222) reflection clearly indicated the cubic indium oxide formation. The film growth rate decreased with increased O_2_ concentration resulting in a much thicker ITO critical thickness (amorphous to polycrystalline transition thickness) for the 0 sccm RF sputtered films. The overall film is mixed phase crystalline and amorphous in nature. The noise is due to the ultra-thin porous and amorphous film left once the top crystalline film is etched off. This is not observed in the 0.4 and 1 sccm films because the increased oxygen composition results in reduced growth rates giving films that are more crystalline in nature with a much thinner critical thickness. 

The lattice parameters and lattice distortion are seen to vary closely with oxygen concentration in the sputter chamber and the length of the etching process. The different oxygen–argon ratios sputtered ITO films have different etching behaviors, which then effected their electrical and optical properties. During the etching process, the crystal lattice of ITO films is degraded due to the exchange of bonds between indium oxide with HCl and HNO_3_. This means that the indium oxide In–O and H–Cl bonds are substituted by In–Cl, In-(NO_3_)_3_ and O–H in the ITO surfaces [49]. These kinds of reactions may reduce the oxygen concentrations and distort structural long range order of the ITO films. This was reflected in the variation of electrical and optical properties of the ITO films with etching time. As no evidence of tin phases were detected, it can be concluded the reactions involving tin phases have negligible effect on the overall etch rates described in this study. 

It is interesting to note that the ITO films processed at the 0.4 sccm oxygen flow rate presented the greatest resistance to acid etching in addition to exhibiting above moderate electrical and optical properties. These films show potential as candidate materials for encapsulation of PV devices or transparent conducting electrodes for varied application in acid-rich environment. However, further research into optimization of anti-acid (acid resistant) ITO films will be required before the material can be implemented in commercial PV devices.

Usually ITO is sputtered in varied combinations of reactive gas environments of argon with oxygen, hydrogen and nitrogen [40]. The oxygen ambient has been shown to be an important parameter to control electrical and optical properties. The highest oxygen concentrations enhance the transmission property and the oxygen deficient nature (oxygen vacancies) increased the electrical conductivity of the ITO thin films [47,48]. Hence, a sufficient amount of oxygen concentration can improve the opto-electronic performance of ITO thin films. High-quality ultra-thin ITO films are a needed significant step towards the realization and possible commercialization of plasmonic-based a-Si:H thin-film PV devices [15,24,36,37,38]. These devices have a potential to transform the thin-film based solar cells industry due to their low cost and ease of fabrication. In addition, plasmonic-enhanced PV has the potential to exhibit sophisticated light management schemes enabling unprecedented control over the trapping and propagation of light within the active region of the PV device [15], which would be expected to result in record-high device solar energy conversion efficiencies.

## 5. Conclusions

In this study, ultra-ITO thin films have been RF sputter deposited using different oxygen flow rates and chemical shaving is performed at room temperature for different time periods. The thicknesses of the films are altered as a result from 89 nm to 22 nm. In-between each etching process cycle, the films were characterized for both electrical and optical properties. Generally, the transmittance of the ITO films was observed to increase with decreasing film thickness, while the electrical properties were observed to degrade for the same films. This was attributed to the distortion of the In–O lattice long-range order due to the reduction reaction between the ITO and the etchants (acids). The novel method of chemical shaving further investigated here, is a simple and low-cost method with the potential to produce low loss and highly conductive ultra-thin and acid resistant ITO films for applications ranging from PV devices transparent electrodes to anti-acid materials. Using this method, ultra-thin ITO films with record low resistivity values (as low as 5.83 × 10^−4^ Ω·cm) were obtained and the optical transmission is generally high in the 300–1000 nm wavelength region for all films. The etching rate strongly depends on the oxygen concentrations of RF sputtered ITO films as well as on the post process annealing. This processing has an effect on the oxygen vacancies densities even for the 0 sccm O_2_ films. Surface roughness increased as the concentration of oxygen increased as expected. The etching reactions are simple redox reaction, hence the rates should increase with increases in O_2_ concentration especially for non-stoichiometric films with distorted ITO matrix. The etch rate, preferred crystal lattice growth plane, d-spacing and lattice distortion were also observed to be highly dependent on the nature of growth environment for RF sputter deposited ITO films.

## Abbreviations

4-PP	Four Point Probe
AFM	Atomic Force Microscopy
a-Si:H	Hydrogenated Amorphous Silicon
AZO	Aluminum doped Zinc Oxide
c-Si	Crystalline Silicon
DC	Direct Current
FTO	Fluorine doped-Tin oxide
ITO	Indium Tin Oxide
PV	Photovoltaic
RF	Radio Frequency
SCCM	Standard Cubic Centimeters per Minute (flow unit)
SLG	Sodaline Glass
SnO_2_	Tin Oxide
TCOs	Transparent Conducting Oxides
VASE	Variable Angle Spectroscopic Ellipsometry
XRD	X-ray Diffraction
ZnO	Zinc Oxide
